# The Steroid Metabolome in the Isolated Ovarian Follicle and Its Response to Androgen Exposure and Antagonism

**DOI:** 10.1210/en.2016-1851

**Published:** 2017-02-23

**Authors:** Marie Lebbe, Angela E. Taylor, Jenny A. Visser, Jackson C. Kirkman-Brown, Teresa K. Woodruff, Wiebke Arlt

**Affiliations:** 1 Institute of Metabolism and Systems Research, University of Birmingham, Birmingham B15 2TT, United Kingdom; 2 Division of Reproductive Science in Medicine, Department of Obstetrics and Gynecology, Feinberg School of Medicine, Northwestern University, Chicago, Illinois 60611; 3 Department of Internal Medicine, Erasmus MC, 3015 CN Rotterdam, The Netherlands; 4 Tommy’s National Centre for Miscarriage Research, Birmingham Women’s Hospital NHS Foundation Trust, Birmingham B15 2TH, United Kingdom; 5 Centre for Endocrinology, Diabetes and Metabolism, Birmingham Health Partners, Birmingham B15 2TH, United Kingdom

## Abstract

The ovarian follicle is a major site of steroidogenesis, crucially required for normal ovarian function and female reproduction. Our understanding of androgen synthesis and metabolism in the developing follicle has been limited by the sensitivity and specificity issues of previously used assays. Here we used liquid chromatography–tandem mass spectrometry to map the stage-dependent endogenous steroid metabolome in an encapsulated *in vitro* follicle growth system, from murine secondary through antral follicles. Furthermore, follicles were cultured in the presence of androgen precursors, nonaromatizable active androgen, and androgen receptor (AR) antagonists to assess effects on steroidogenesis and follicle development. Cultured follicles showed a stage-dependent increase in endogenous androgen, estrogen, and progesterone production, and incubations with the sex steroid precursor dehydroepiandrosterone revealed the follicle as capable of active androgen synthesis at early developmental stages. Androgen exposure and antagonism demonstrated AR–mediated effects on follicle growth and antrum formation that followed a biphasic pattern, with low levels of androgens inducing more rapid follicle maturation and high doses inhibiting oocyte maturation and follicle growth. Crucially, our study provides evidence for an intrafollicular feedback circuit regulating steroidogenesis, with decreased follicle androgen synthesis after exogenous androgen exposure and increased androgen output after additional AR antagonist treatment. We propose that this feedback circuit helps maintain an equilibrium of androgen exposure in the developing follicle. The observed biphasic response of follicle growth and function in increasing androgen supplementations has implications for our understanding of polycystic ovary syndrome pathophysiology and the dose-dependent utility of androgens in *in vitro* fertilization settings.

## Teaser

Steroid metabolome analysis by mass spectrometry in the isolated ovarian follicle revealed early developmental capacity for androgen synthesis, which was upregulated by androgen receptor antagonists.

Female reproductive health relies on the proper development of the follicle, the fundamental unit of the ovary. As waves of follicles grow, they produce sex steroid hormones that regulate maturation in an autocrine/paracrine manner, supply endocrine feedback that sets the tempo of each reproductive cycle, prepare the reproductive tissues for pregnancy, and regulate bone, cardiovascular, and metabolic health. Many elegant studies have evaluated androgen production in various follicle culture and *in vivo* settings (1–6). We have extensively validated a method to study encapsulated *in vitro* ovarian follicle growth (eIVFG) from mouse, bovine, goat, canine, nonhuman primate, and human biomaterials, all of which result in mature eggs or embryos (7–15). Steroid hormone measurements in this culture system provided valuable information but relied on immunoassays (7, 16). This latter technology is hampered by intrinsic problems of sensitivity and specificity, especially in the presence of low steroid concentrations (17), such as the production of androgens by individual preantral growing follicles in culture. Modern mass spectrometry–based steroid analysis overcomes these challenges (18) but has not yet been applied to the developing follicle. Here we studied endogenous basal steroid production in isolated ovarian follicles by liquid chromatography–tandem mass spectrometry (LC-MS/MS), employing a murine eIVFG system.

Another advantage of eIVFG is the possibility of directly studying the dose- and stage-dependent effects of exogenous factors on individual follicle development and function. Manipulating the local or endocrine microenvironment of the growing follicle may also phenocopy certain aspects of human ovarian disease (19). With use of eIVFG, testosterone directly increases survival and growth of macaque secondary follicles, supporting the notion that androgens regulate follicle dynamics (20). Indeed, androgen action is essential for preantral follicle development, as initially demonstrated by global androgen receptor (AR) knockout models and mirrored by the granulosa cell–specific AR knockout mice, in which females are subfertile and have reduced follicle development, altered gonadotrophin regulation, decreased ovulation rates, and poor oocyte quality (21–26). Recent work has shown that nuclear and extranuclear AR-mediated signaling pathways are crucially involved in promoting follicle growth and survival (27).

These fundamental studies are important because alterations in androgen homeostasis in women may result in infertility and anovulation. In clinical conditions of androgen excess, as observed in women with polycystic ovary syndrome (PCOS), follicle development is arrested, leading to chronic anovulation and subfertility (28, 29). The dysfunctional follicle phenotype may relate to excess androgen exposure during critical developmental stages, as demonstrated by studies in mice showing that prepubertal androgen exposure leads to follicular arrest and increased follicular atresia (30). Similarly, in nonhuman primates, *in vivo* exposure to exogenous androgens in early gestation results in PCOS-like ovarian dysfunction in the adult offspring, manifesting with follicle excess, oligomenorrhea, and hyperandrogenemia (31, 32). Although androgen excess is deleterious for follicle development, androgen deficiency might equally alter follicle maturation. In assisted reproductive clinics, androgen supplementation, either with the androgen precursor dehydroepiandrosterone (DHEA) or with testosterone, is widely used to improve follicular development and fertility in women with diminished ovarian reserve (33–35).

Here we have used the murine eIVFG system and steroid analysis by LC-MS/MS to comprehensively map the stage-dependent endogenous steroid metabolome of the follicle during development and to directly examine the dose-dependent effects of the nonaromatizable potent androgen 5*α*-dihydrotestosterone (DHT), the sex steroid precursor DHEA, and the selective AR antagonist enzalutamide (MDV) on follicular function and steroidogenesis.

## Methods

### Murine encapsulated *in vitro* follicle culture

CD1 mice were housed and bred in a temperature- and light-controlled (12-hour light, 12-hour dark cycle) environment and were provided with unrestricted access to water and chow (PicoLab Mouse Diet 20; Sandown Scientific, Hampton, UK) in the Biomedical Services Unit at the University of Birmingham. Nonweaned pups (days 15 to 17) were culled by cervical dislocation before dissection for excision of ovarian tissue. The euthanasia procedure was conducted in accordance with current UK Home Office regulations in accordance with the UK Animal (Scientific Procedures) Act 1986 and was covered by the generic breeding license of the Biomedical Services Unit. Ovaries were transported in L-15 GlutaMAX medium (Thermo Fisher Scientific, Loughborough, UK) supplemented with 1% fetal bovine serum (FBS; Sigma-Aldrich, Gillingham, Dorset, UK) and 0.5% penicillin/streptomycin (Thermo Fisher Scientific) in a carrier-incubator at 37°C. After transport, ovaries were transferred to a dish containing L-15 medium supplemented with 0.1% DNase I (Lorne Laboratories Limited, Reading, UK) and 0.1% Liberase TM (Roche Life Science, West Sussex, UK) and were placed on a shaker in a 37°C 6% CO_2_ incubator for 35 to 40 minutes.

After the addition of 10% FBS, multilayered secondary follicles (diameter, 150 to 180 μm) were mechanically isolated employing insulin-gauge needles under a dissection scope. Follicles were placed in a maintenance medium containing minimal essential medium (*α*-MEM GlutaMAX; Life Technologies Ltd, Paisley, UK) supplemented with 1% FBS and 0.5% penicillin/streptomycin for 2 to 3 hours in a 37°C 6% CO_2_ incubator.

Follicles were individually encapsulated in 0.5% alginate (NovaMatrix, Sandvika, Norway) and were allowed to crosslink in a calcium solution for 2 minutes, as previously described (7). Alginate beads were transferred to a 96-well plate, with each well containing 100 μL culture medium consisting of *α*-MEM GlutaMAX supplemented with 3 mg/mL bovine serum albumin (BSA; MP Biomedicals, Leicester, UK), 10 mIU/mL recombinant follicle-stimulating hormone (FSH; Gonal-f; Merck Serono, Feltham, UK), 1 mg/mL bovine fetuin (Sigma-Aldrich), and 5 μg/mL insulin, 5 μg/mL transferrin, and 5 μg/mL selenium (Sigma-Aldrich).

For the treatment conditions, culture medium was supplemented with 25 or 50 nM DHT (Sigma-Aldrich); 100, 200, or 500 nM DHEA (Sigma-Aldrich); and 10 or 25 nM estradiol (E2) (Sigma-Aldrich). The steroid concentrations used were based on published dose-response experiments (27, 36). For AR blockade, MDV (Axon Medchem, Groningen, The Netherlands) was used at the dose of 1 μM on the basis of its half maximal inhibitory concentration value (37).

After plating, encapsulated follicles were imaged using a Nikon Eclipse TE300 light microscope (Leica, Nikon, UK) with 10× phase objective. Follicles with intact alginate beads and with preserved integrity of the oocyte and somatic cell compartment were selected for culture. Follicles were cultured for 6 days in a 37°C 6% CO_2_ incubator. Media changes (50 μL) were performed on alternate days, with fresh steroids at the initial concentration for the treatment conditions as well as repeated imaging. Images were analyzed using ImageJ Software (National Institutes of Health, Bethesda, MD).

Follicle sizes were obtained by averaging two perpendicular measurements of follicle diameter. The movement of the oocyte to an eccentric position with the appearance of a fluid-filled space determined the presence of an antrum. Follicles were classified as nonviable when the oocyte or somatic compartment appeared shrunken or dark, when their interphase was compromised, or when the alginate bead was disrupted. Only surviving follicles were included in the data analysis.

### *In vitro* follicle maturation

After the 6-day culture period, follicles were retrieved from the alginate bead using alginate lyase (Sigma-Aldrich) and were transferred to a maturation medium composed of *α*-MEM GlutaMAX, 10% FBS, 1.5 IU/mL human chorionic gonadotropin (Sigma-Aldrich), and 5 ng/mL epidermal growth factor (BD Biosciences, Oxford, UK) for 16 hours at 37°C, 6% CO_2_, as previously described (7). Oocytes were then denuded from the surrounding cumulus cells by treatment with 0.3% hyaluronidase (Sigma-Aldrich) and gentle aspiration. The oocytes were classified as mature, or metaphase II, when a polar body was visible in the perivitelline space. Healthy oocytes that had not resumed meiosis were classified as immature.

### Steroid analysis by LC-MS/MS

Pooled follicle culture supernatant (from 30 to 100 follicle incubations) was placed in silanized glass tubes, and 20 μL of internal standard was added. Three milliliters of methyl *tert*-butyl-ether was added to each sample, followed by vortexing and freezing for 1 hour. The upper organic phase was transferred to a 96-well plate using glass Pasteur pipettes, followed by evaporation under nitrogen at 55°C. Samples were reconstituted with 125 μL methanol:water mixture (50:50) and were frozen at −20°C before analysis. Steroids were quantified by LC-MS/MS using a Waters Xevo mass spectrometer with an Acquity UPLC system with the following settings: electrospray ionization source with capillary voltage at 4.0 kV, temperature source at 150°C, and a desolvation temperature of 500°C.

Steroid identification was based on an identical retention time and two identical mass transitions when compared with authentic reference compounds. Quantification was performed relative to a calibration series (0, 0.5 to 250 ng/mL of each steroid) with an appropriate internal standard steroid, as previously described (38), and was appropriately validated, including determination of the lower limits of detection (LLOD) and quantification (LLOQ) (Table 1). Steroid concentrations above the steroid-specific LLOQ were considered accurately quantified; steroid concentrations below the steroid-specific LLOQ but above the respective LLOD were described as detectable. All measurements were performed in triplicate except for treatment conditions DHT + MDV and DHEA 100 nM, which were assessed in duplicate because of a shortage of biological material.

**Table 1.** **Lower Limits of Quantification and Detection of Liquid Chromatography–Tandem Mass Spectrometry Assay for Multisteroid Profiling**

**Steroid**	**LLOQ** **(nmol/L)**	**LLOD** **(nmol/L)**
Prog	1.6	0.8
DHEA	1.7	0.9
A’dione	1.8	0.8
Testosterone	2.0	0.9
DHT	4.5	1.7
E1	4.0	1.9
E2	4.0	1.8

LLOQ and LLOD were calculated from calibration series experiments employing steroid-spiked cell culture media. LLOQ was defined as a detectable signal with a signal/noise ratio of more than 10:1 and with a signal variation of <20%. LLOD was defined as the lowest detectable concentration with a signal/noise ratio of more than 3:1 and with a signal variation of <20%.

Abbreviations: A'dione, androstenedione; E1, estrone; Prog, progesterone.

### Messenger RNA expression analysis

At the end of culture, we pooled 18 to 30 follicles for each experimental condition, which were immediately flash frozen in liquid nitrogen. RNA was purified from the follicles using the RNeasy Micro Kit (Qiagen, Manchester, UK). RNA quality and quantity were assessed employing NanoDrop technology (ND-1000; Thermo Fisher Scientific) and High Sensitivity R6K ScreenTape System (Agilent, Cheshire, UK). RNA was diluted to a concentration of 50 to 100 ng/μL. RNA was reverse transcribed to complementary DNA (cDNA) using an AccuScript High Fidelity 1st Strand cDNA Synthesis Kit (Agilent Technologies) according to the instructions of the manufacturer. Messenger RNA (mRNA) expression levels were assessed by quantitative polymerase chain reaction using an ABI sequence detection system (Perkin-Elmer Applied Biosystems, Warrington, UK). All analyses were assessed in 10-μL final volume in reaction buffer, containing 2 X Taqman Universal PCR Master Mix (5.0 μL; Thermo Fisher Scientific), probe-primer mix for the target gene (0.5 μL), and 4.5 μL cDNA (100 ng) (39). All reactions were normalized against the housekeeping genes 18S ribosomal RNA and ribosomal protein L18 (Rpl18) ribosomal RNA. Data were expressed as Δcycle threshold (CT) values [ΔCT = (CT of target gene) − (CT of housekeeping gene)].

### Statistical analysis

Statistical analysis was performed with Prism 6 (GraphPad) software, using one-way analysis of variance with a *post hoc* Tukey test to compare follicle growth and oocyte size between the different treatment groups. Contingency analysis by Fisher’s exact test was used for survival, antrum formation, and oocyte maturation status. Independent *t* tests were used to compare steroid quantifications and ΔCT values between control and treatment conditions. Matched or repeated measurements were analyzed using paired *t* tests, and unpaired *t* tests were used for independent measurements. All studies were performed in at least three independent experiments unless otherwise specified.

## Results

### Endogenous steroid synthesis in the developing follicle

**Figure 1.**
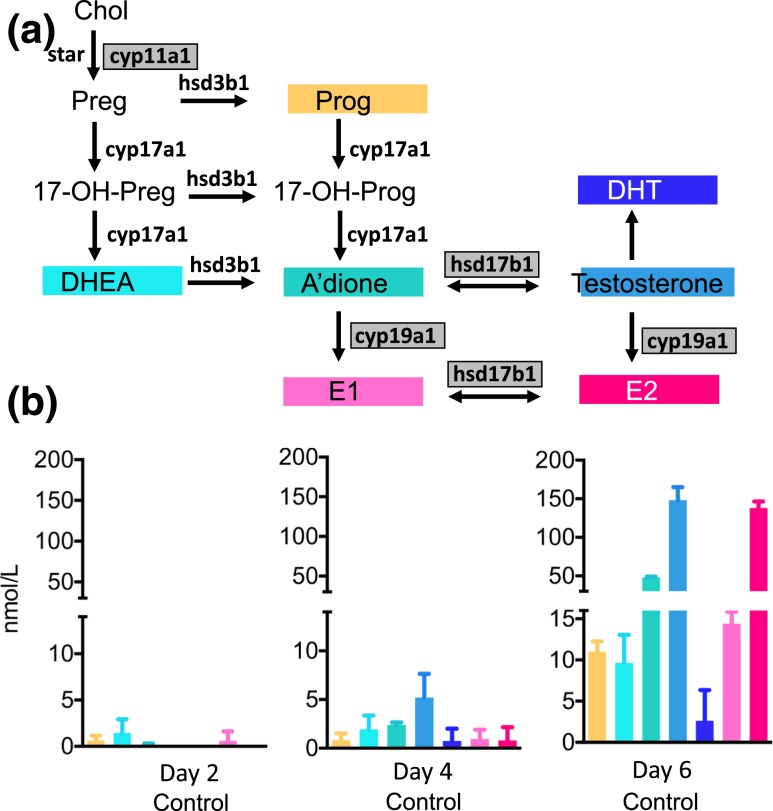
Steroid hormone production by the cultured follicles, measured by LC-MS/MS, in follicle culture medium pooled from incubations with 72 to 105 individual follicles at days 2, 4, and 6 of culture and normalized to 100 follicles. Data are expressed as mean ± standard deviation. (a) The steroidogenic pathway and the murine steroidogenic enzymes involved in the conversions. Gray-shaded boxes indicate significant upregulation of mRNA expression on day 6 of follicular development. (b) Endogenous steroid production in the isolated follicle. Panels C through E show intrafollicular steroidogenesis in the untreated follicle and after incubation with DHT 25 nM without and with the addition of the AR antagonist MDV(1 μM).17-OH-Prog, 17-hydroxyprogesterone; Chol, cholesterol; CYP17a1, 17-hydroxylase; hsd3b1, 3*β*-hydroxysteroid dehydrogenase; StAR, steroidogenic acute regulatory protein.

We used a murine eIVFG system to assess stage-dependent steroidogenesis in the developing follicle using mass spectrometry–based multisteroid profiling optimized for highly sensitive and specific detection of sex steroids and their precursors [Fig. 1(a)]. At day 2 of culture, we detected progesterone (Prog) and the sex steroid precursors DHEA, androstenedione (A’dione), and estrone (E1) at levels close to the lower limit of detection (0.5 to 2.0 nmol/L) [Fig. 1(b)]. At day 4, the androgen precursors DHEA and A’dione as well as bioactive testosterone were generated in quantifiable amounts, and 17*β*-estradiol became detectable. At day 6 of culture, Prog synthesis increased significantly (*P* < 0.001 vs day 4) to the quantifiable range, and we observed a significant surge in active sex hormones, including testosterone (*P* < 0.001), DHT (*P* = 0.04), and 17*β*-estradiol (*P* < 0.001) [Fig. 1(b)].

Corresponding to the increasing production of active sex steroids across follicle development, steroid enzyme mRNA also increased in a stage-dependent fashion. A significant (*P* < 0.01) increase in 17*β*-hydroxysteroid dehydrogenase type 1 was noted by day 6 (Supplemental Table 1). This enzyme catalyzes the conversions of A’dione to testosterone and E1 to E2 and is FSH responsive (40). Concurrent with follicle maturation, we detected significantly increased transcription (*P* < 0.05) of the FSH-regulated *CYP19a1* gene encoding aromatase, the enzyme responsible for the conversion of androgens to estrogens (Supplemental Table 1). Consistent with the increasing generation of Prog detected by mass spectrometry, mRNA expression analysis showed increased transcription of the side-chain cleavage enzyme CYP11a1 (*P* < 0.01) [Supplemental Table 1; Fig. 1(a)].

### Effect of exogenous androgen exposure and antagonism on follicular development and steroidogenesis

We next examined the direct effects of androgen supplementation on follicle morphology, oocyte development, and steroid synthesis in the isolated follicle. For these studies, we delivered exogenous DHT to secondary follicles. DHT is the most potent androgen, which, in contrast to testosterone, cannot be converted to estrogens by aromatase activity. To determine AR-mediated androgen effects, we used AR blockade by administration of the highly selective AR antagonist MDV, isolated and in combination with DHT. Individual follicles were imaged at days 0, 2, 4, and 6 of culture to study follicle growth by measuring follicle diameters, antrum formation, and follicle survival. Oocyte quality was assessed following *in vitro* maturation.

**Figure 2.**
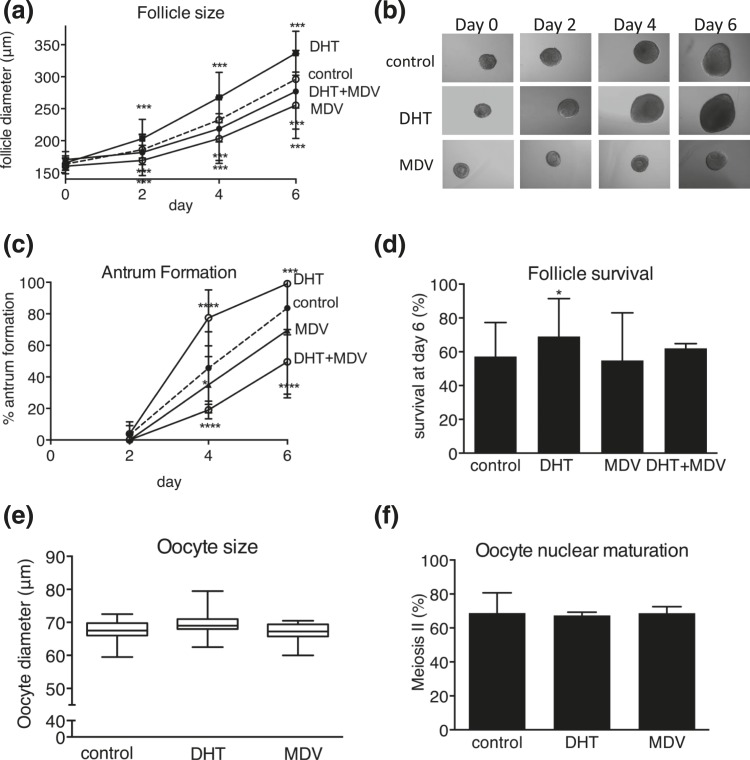
Effect of DHT 25 nM, AR antagonist MDV, and combined DHT + MDV treatment versus control (dotted lines) on follicle and oocyte morphology during culture, with regard to (a) follicle size, (b) light microscopic images, (c) antrum formation rate, (d) follicle survival, (e) oocyte size, and (f) oocyte nuclear maturation status. Data from 57 to 170 follicles per condition are expressed as mean ± standard deviation for diameter, antrum formation, survival, and oocyte meiotic status. Oocyte size is presented in box and whisker plots, with boxes representative of the interquartile range and whiskers representative of the 5th and 95th percentiles. *P < 0.05; ***P < 0.001; ****P < 0.0001.

DHT-treated follicles were significantly growth advanced at all stages of follicular development [Fig. 2(a) and 2(b)]. Conversely, MDV- and DHT + MDV–treated follicles were growth restricted compared with control follicles [Fig. 2(a) and 2(b)]. DHT supplementation resulted in significant acceleration of the preantral to antral follicle transition (*P* < 0.0001), with a higher total number of follicles reaching the antral stage at day 6 (*P* < 0.001) [Fig. 2(c)]. By contrast, MDV- and DHT + MDV–treated follicles showed evidence of delayed antrum formation (*P* < 0.05 at day 4 for MDV follicles and *P* < 0.0001 at days 4 and 6 for DHT + MDV follicles) [Fig. 2(c)]. DHT-treated follicles had a significantly increased survival rate compared with control follicles; survival of MDV- and DHT + MDV–treated follicles did not differ from that of controls [Fig. 2(d)]. Neither DHT exposure nor MDV treatment significantly regulated oocyte size [Fig. 2(e)] or nuclear maturation [Fig. 2(f)] compared with control follicles. These results document the crucial role of AR-mediated androgen action in achieving optimal follicle growth, antrum formation, and protection from atresia.

**Figure 3.**
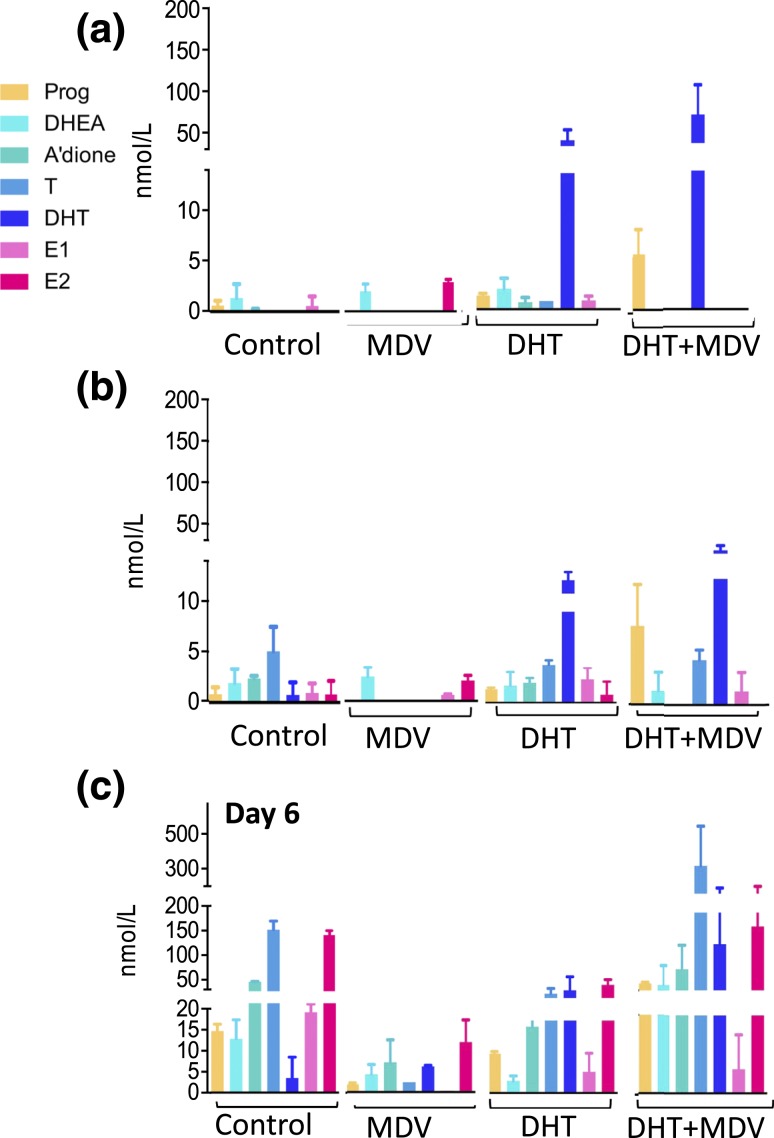
Intrafollicular steroidogenesis in the untreated follicle and after incubation with the AR antagonist MDV (1 μM), with DHT 25 nM, and with co-incubation of DHT 25 nM and MDV 1 μM, across follicular development. (a–c) Panels represent days 2, 4, and 6 of culture, respectively. The color coding refers to Fig. 1(a). Steroids were measured by LC-MS/MS in pooled follicle culture medium (57 to 105 follicles) and normalized to 100 follicles. Data are expressed as mean ± standard deviation.

Exposing follicles to exogenous DHT did not change steroidogenesis at days 2 and 4 [Fig. 3(a) and 3(b)] but significantly decreased endogenous androgen synthesis at day 6, calculated as the sum of DHEA, A’dione, and testosterone (80 ± 15 nmol/L vs 208 ± 10 nmol/L in controls; *P* < 0.01) [Fig. 3(c)]. *Vice versa*, administration of DHT in combination with the AR antagonist MDV resulted in a significant upregulation of follicular androgen synthesis at day 6 (490 ± 256 nmol/L with DHT + MDV vs 80 ± 15 nmol/L with DHT alone; *P* < 0.05) [Fig. 3(c)]. These differences in androgen production were not mirrored by significant changes in steroidogenic enzyme expression at the mRNA level (data not shown). These results suggest that *in vitro* cultured follicles are capable of autonomously adapting endogenous androgen synthesis in response to changes in AR activation status, possibly indicating an intrafollicular AR-mediated autocrine feedback circuit involved in steroidogenesis. Interestingly, the addition of MDV alone resulted in significantly decreased steroid output at days 4 and 6 (Fig. 3), which suggests that the observed intrafollicular feedback circuit becomes activated only after induction by endogenous or exogenous androgen exposure.

DHT treatment decreased E2 synthesis at day 6 (77 ± 10 nmol/L vs 138 ± 8 nmol/L in controls; *P* < 0.01) and had no effect on Prog production (10 ± 0.5 nmol/L vs 11 ± 1 nmol/L in controls; not significant). DHT + MDV supplementation tended to increase E2 synthesis at day 6 (169 ± 75 nmol/L vs 77 ± 10 nmol/L with DHT alone; not significant) and significantly increased Prog production (45 ± 2 nmol/L vs 10 ± 0.5 nmol/L with DHT alone; *P* < 0.01).

When calculating the androgen/estrogen ratio [(A’dione + testosterone)]/(E1 + E2)] to assess aromatase activity (5, 41), we found that DHT and DHT + MDV follicles maintained the balance observed in control follicles (DHT vs controls on day 6: 0.9 ± 0.2 vs 1.3 ± 0.01, respectively, *P* = 0.2; DHT + MDV vs controls on day 6: 2.4 ± 0.5 vs 1.3 ± 0.01, respectively, *P* = 0.1).

### The androgen precursor DHEA is converted to active sex steroid in the developing follicle

**Figure 4.**
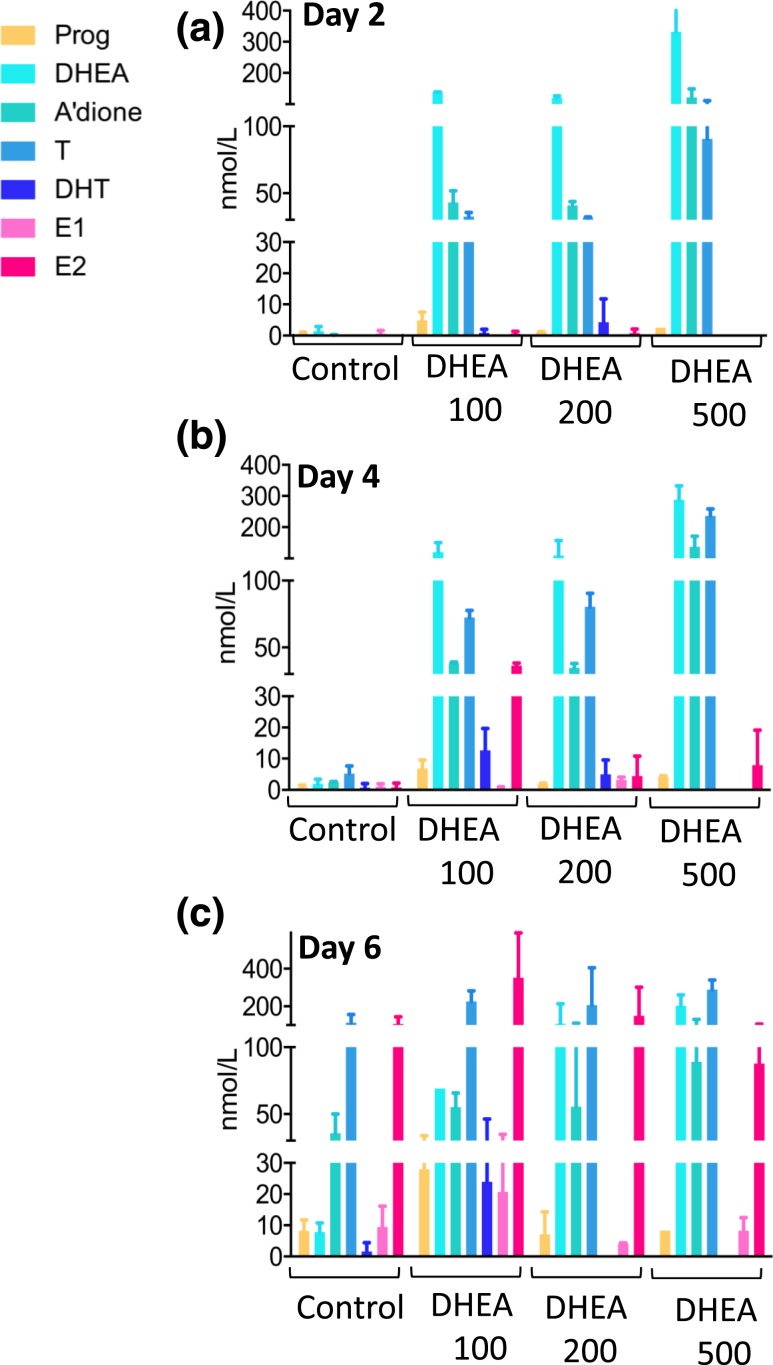
Effect of increasing concentrations of DHEA supplementation (100, 200, and 500 nM) on follicular steroid hormone production compared with control conditions across follicular development. (a–c) Panels represent days 2, 4, and 6 of culture, respectively. The color coding refers to Fig. 1(a). Steroids were measured by LC-MS/MS in pooled follicle culture medium (30 to 105 follicles) and were normalized to 100 follicles. Data are expressed as mean ± standard deviation.

Because the secondary follicles synthetize appreciable levels of steroid hormones in the second half of the *in vitro* culture, we used the addition of the sex steroid precursor DHEA as a probe to further examine the stage-dependent steroidogenic capacity of the follicle. Steroid profiling by LC-MS/MS revealed that DHEA was actively converted by the follicle at all time points, including the immature stage (day 2) when endogenous steroidogenesis in control follicles was not quantifiable [Fig. 4(a)]. Supplementation with 100 nM DHEA revealed high capacity for downstream androgen generation (A’dione, testosterone, and DHT) and high levels of conversion to estrogens at day 4 [Fig. 4(b)], which appeared further enhanced by day 6 [Fig. 4(c)]. When increasing DHEA concentrations to 200 nM and 500 nM, we observed a gradual loss of appreciable generation of DHT from testosterone alongside a decrease in estrogen production, which became significant at day 6 of 500 nM DHEA (*P* < 0.05) [Fig. 4(c)]. At mRNA level, incubation with DHEA resulted in a significant (*P* < 0.05) downregulation of *CYP19a1* mRNA expression compared with control follicles at all concentrations used (ΔCT CYP19a1: DHEA 0 nM, 12.3 ± 1.3; DHEA 100 nM, 15.9 ± 0.8; DHEA 200 nM, 14.1 ± 0.4; DHEA 500 nM, 14.9 ± 0.2).

When calculating the androgen/estrogen ratio [(A’dione + testosterone)/(E1 + E2)] to assess aromatase (CYP19a1) activity (5, 41), we found that DHEA 100 nM maintained the balance observed in control follicles (DHEA 100 nM vs controls on day 6: 0.9 ± 0.3 vs 1.3 ± 0.01, respectively; *P* = 0.3), whereas higher DHEA concentrations significantly increased the androgen/estrogen ratio (DHEA 200 nM, 1.7 ± 0.09, *P* < 0.01 vs controls; DHEA 500 nM, 4.3 ± 1, *P* < 0.05; day 6), indicative of an androgenic intrafollicular milieu.

Thus, using exogenous DHEA administration as a probe, we found that earlier stages of the developing follicle were capable of active androgen generation and that increased exposure to DHEA resulted in inhibition of aromatase activity and, consequently, estrogen production.

### Effects of increasing concentrations of exogenous androgens and estrogens on follicular development

Next, we looked at the impact of the androgen precursor DHEA on follicular development and oocyte maturation. We showed that DHEA was converted by the follicles to androgens and subsequently estrogens. Therefore, we compared the effects observed after DHEA stimulation with the impact of increasing doses of nonaromatizable DHT and biologically active estrogen, E2, to dissect effects due to androgens vs estrogens in a potentially distinct fashion.

**Figure 5.**
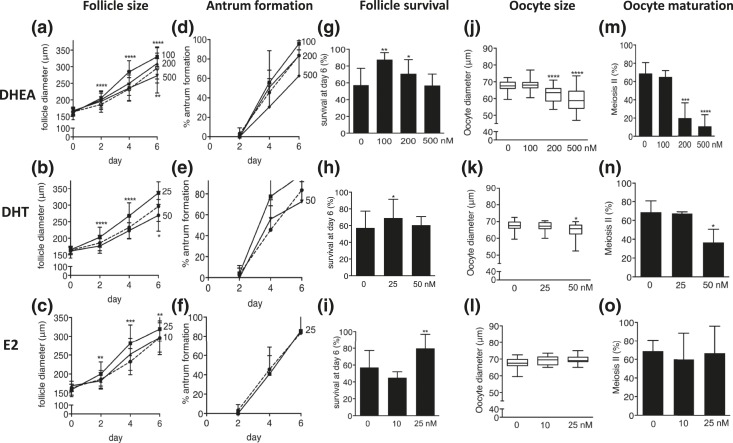
Effect of increasing concentrations of DHEA supplementation (100, 200, and 500 nM) and increasing concentrations of DHT (25 and 50 nM) and E2 (10 and 25 nM) supplementation versus control (dotted lines) on follicle and oocyte biology during culture, with regard to (a–c) follicle size, (d–f) antrum formation rate, (g–i) follicle survival, (j–l) oocyte size, and (m–o) oocyte nuclear maturation status. Data (n = 31 to 170 follicles/condition) are expressed as mean ± standard deviation for diameter, antrum formation, survival, and meiotic maturation. Oocyte size is presented in box and whisker plots, with boxes representative of the interquartile range and whiskers representative of the 5th and 95th percentiles. *P < 0.05; **P < 0.01; ***P < 0.001; ****P < 0.0001.

Follicle size, reflective of follicular growth, was enhanced by DHEA 100 nM and DHT 25 nM [Fig. 5(a) and 5(b)]; increasing androgen exposure to DHEA 200 nM neutralized this effect, and a further increase to DHEA 500 nM and exposure to DHT 50 nM showed the opposite effect, with a significant reduction in follicle size [Fig. 5(a) and 5(b)]. The higher dose of E2 (25 nM) increased follicle size significantly, whereas 10 nM of E2 had no effect [Fig. 5(c)].

Antrum formation was significantly enhanced by DHT 25 nM, whereas increasing DHT to 50 nM reverted this effect [Fig. 5(e)]. DHEA 100 nM appeared to have a beneficial effect on antrum formation, whereas higher concentrations had an adverse effect, though the differences failed to reach statistical significance [Fig. 5(d)]. E2 had no effect on antrum formation [Fig. 5(f)].

Follicle survival rates increased significantly with exposure to DHEA 100 and 200 nM and DHT 25 nM. This effect was lost when DHEA was increased to 500 nM and DHT was increased to 50 nM. By contrast, E2 at 10 nM yielded no discernible effect on follicle survival, whereas a significant increase was observed after increasing E2 to 25 nM [Fig. 5(g–i)].

Oocyte size was significantly reduced by the higher concentrations of DHEA (200 and 500 nM) and the higher DHT concentration (50 nM) [Fig. 5(j) and 5(k)]. By contrast, E2 exposure had no effect on oocyte size [Fig. 5(l)]. These findings were completely mirrored when assessing oocyte nuclear maturation, which was significantly decreased by higher androgen concentrations but was not affected by estrogen administration [Fig. 5(m–o)].

Taken together, although moderate androgen levels exerted beneficial effects on follicle growth, survival, and antrum formation, increasing bioactive androgen caused poor oocyte quality and negative effects on follicle growth and antrum formation. Increasing estrogen exposure enhanced follicle growth and survival, with no effect on antrum formation or oocyte quality.

## Discussion

Although previous studies have proven the importance of androgen action in follicular development, our study extends this knowledge by approaching the follicle as a coordinated steroidogenic unit. We report simultaneous quantitative analysis of multiple steroids in the developing murine follicle under physiological conditions and in the presence of androgen exposure and antagonism, with highly sensitive and specific multisteroid profiling by tandem mass spectrometry. We demonstrated that the growing follicle has the capacity for active sex steroid synthesis at all developmental stages examined and provided evidence for the existence of an intrafollicular AR-responsive feedback circuit that dynamically regulated androgen synthesis in an autonomous fashion. We confirmed the beneficial effects of low-dose androgen supplementation to the growing follicle and described an AR-mediated facilitating role in antrum formation. Finally, we observed that gradually increasing androgen concentrations resulted in follicle developmental arrest, characterized by suppressed oocyte maturation, follicular growth stagnation, and decreased estrogen synthesis.

We reported a quantitative multisteroid metabolome of the developing follicle, indicative of FSH-stimulated endogenous production of androgens, estrogens, and progestins, consistent with the current knowledge of follicular steroid production and *in vivo* hormone dynamics (42). Androgen secretion was quantifiable around day 4, which corresponds to antrum formation and gonadotrophin responsiveness. Estrogen biosynthesis increased sharply between days 4 and 6 of culture, as the follicle reached ovulatory maturity. At day 6, Prog secretion started to increase, as expected, in preparation for luteinization. The FSH-dependent steroidogenic enzymes 17*β*-hydroxysteroid dehydrogenase type 1 and CYP19a1 significantly increased with ongoing follicle maturation. According to the two-cell, two-gonadotrophin hypothesis, luteinizing hormone (LH) stimulates A’dione production by theca cells, which provides a substrate for estrogen biosynthesis by granulosa cells (43). In the culture system used, however, maturing follicles produced significant amounts of E2 in an LH-free and serum-free medium. This means that follicular cells are able to constitutively produce androgens in the absence of LH. Paracrine theca cell LH-independent androgen production is known to occur under the influence of insulin (44, 45), present in physiological amounts in the culture medium used. Expression of steroidogenic acute regulatory protein, a theca cell marker (46), remained stable during follicle culture, which suggests that *de novo* theca cell formation was limited and that granulosa-theca cell *trans*-differentiation possibly accounts for the observed androgen production. Although the eIVFG system allowed for complete theca cell development, LH was not present; future studies on the effects of increasing doses of LH on androgen production in our system would enhance the translatability of our results.

In our study, LC-MS/MS measured similar (47) or lower (7, 48) concentrations of A’dione and E2 compared with those obtained in similar culture conditions but measured with immunoassays. These differences could be explained by the fact that immunoassays are prone to cross-reactivity, which may lead to falsely increased concentrations (49). A major advantage of our mass spectrometry–based multisteroid profiling assay is the ability to simultaneously measure multiple steroid concentrations in a single assay, whereas immunoassays are limited to one target molecule. Therefore, our validated mass spectrometry approach (50) yielded a state-of-the-art representation of the dynamic endogenous steroid production in murine follicles.

When follicles were exposed to exogenous DHT, the most potent and nonaromatizable androgen, we observed a downregulation of endogenous androgen secretion. This was AR-mediated, as the addition of the selective AR antagonist MDV prompted increased endogenous androgen synthesis. These findings are indicative of a feedback circuit at the level of the follicle, which may provide the homeostatic set point for androgen-AR downstream effects.

We further reported a detailed analysis of AR-mediated androgen effects on the development of the follicle and oocyte. The current results are in line with the previously reported follicular growth-promoting effects of androgens (4, 27, 36, 51) and their roles in protecting from atresia (27) and enhancing follicle survival (20). We showed that the process of antrum formation occurred earlier and to a higher extent in DHT-treated follicles and was impaired in AR-blocked follicles. It was previously shown that follicles grown in antiandrogen serum (4), in the absence of FSH (27) or in steroid-depleted conditions (20), displayed limited antrum formation. Oocyte growth and maturation were not affected by AR agonist (DHT) or antagonist (MDV) treatment in our system. Tarumi *et al.* (52) treated mouse ovarian follicles in culture with a concentration rate of 10^−10^ to 10^−6^ M DHT and found no effect on the capacity of the oocyte to resume meiosis following an ovulatory stimulus. Lenie and Smitz (5) observed no change in oocyte quality when treating mouse follicles *in vitro* with the AR-antagonist hydroxyflutamide or bicalutamide (in a concentration range of 5 nM to 5 μM), and only the highest dose (50 μM) of AR blockade resulted in decreased oocyte meiotic maturation.

Murine steroidogenesis resembles human steroid production but differs slightly in some details; for example, the human CYP17A1 enzyme does not efficiently convert 17-hydroxyprogesterone to A’dione (53), which means that the overwhelming majority of androgen synthesis in humans proceeds through the androgen precursor DHEA. The addition of 100 nM of DHEA had positive effects on follicle growth and survival and did not impair oocyte development, whereas increasing concentrations of DHEA (200 and 500 nM) provoked dysfunctional follicle development, with dose-dependent robust suppression of oocyte growth and maturation, aromatase enzyme activity, estrogen production, and follicular proliferation. Previous studies reported that *in vitro* supplementation of mouse follicles cultured with A’dione at doses >200 nM (54) or 10^−5^ M (55) was associated with decreased meiotic maturation and impaired spindle formation. The toxic effect on the oocyte was attributed to estrogen excess in one study (52) and was inconclusive with regard to its androgen-mediated mechanism in the other study (54). In our study, the detrimental oocyte phenotype in >200 nM of DHEA-treated follicles was clearly attributable to increased provision of active androgens to the follicle generated by conversion of DHEA to testosterone (T) and DHT. The androgen-mediated downregulation of aromatase is in line with reported observations in granulosa (56) and Leydig cells (57, 58). In rats, administration of DHT was accompanied by decreased granulosa cell proliferation (59), suppressed aromatase activity, and reduced E2 production (60). In primates, DHT administration resulted in reduced FSH-stimulated estrogen synthesis (61). DHEA does not mediate its effect by direct binding and transactivation of the AR but exerts androgenic activity only indirectly, after downstream conversion to AR-binding androgens, such as testosterone and DHT. In the context of our experiments with isolated murine ovarian follicles, we used DHEA as a probe for exploring the steroidogenic capacity of the developing follicle, which is more readily achieved by adding substrate than by looking at baseline production only. Employing single-follicle steroid metabolome analysis, we showed that the follicle is capable of downstream conversion of DHEA to active androgen as early as day 2 of follicular development.

Our findings were obtained with experimental androgen concentrations in murine follicles. Therefore, we have to be cautious in translating them to human pathologic follicle development in hypoandrogenic or hyperandrogenic conditions; however, some general implications might hold true. Our results contribute to the scientific foundation for DHEA pretreatment in poor responder women undergoing IVF to improve the developmental quality of the maturing follicles. As others have highlighted before (62, 63), the maturing follicle is subject to a delicate androgen homeostasis, with a clear threshold level. In our study, using a murine model this threshold is DHEA 200 nM, beyond which the beneficial effects of enhanced active androgen generation become deleterious. Although a murine model has limitations in assessing DHEA action (*i.e*., given its limited physiological role in rodents), our results appear to indicate that over-replacement of DHEA in human-assisted reproductive settings might actually harm oocyte quality and become detrimental for follicle growth. This study clearly underlines the need for adequately powered, randomized, controlled trials on DHEA supplementation that takes into account baseline levels of circulating androgens and aims to restore physiological DHEA concentrations in women with low ovarian reserve undergoing fertility workup. Previous studies have shown that daily doses of DHEA (25 to 50 mg) restore physiological serum androgen concentrations from nondetectable baseline concentrations in women with adrenal insufficiency (64–66). Daily doses of ≥75 mg of DHEA will yield supraphysiological androgen concentrations (64). However, these are the doses used by many studies targeting enhanced fertility by DHEA treatment (67, 68), which renders DHEA administration in this context a pharmacological intervention.

Sex steroid production occurred earlier in DHEA-treated follicles than in nonstimulated follicles, indicating that in the presence of steroid substrate, immature follicles are steroidogenically active and capable of androgen synthesis. In women with PCOS, DHEA and A’dione production is increased (69–71), and these circulating androgen precursors are likely to be metabolized by the small preantral PCOS follicles, thereby contributing to intraovarian hyperandrogenism. The intrafollicular feedback circuit we observed, with decreased endogenous androgen synthesis after exogenous DHT and increased androgen production with added AR antagonist, may help to maintain an androgen equilibrium in the follicle, providing steady levels of AR activation during development to maximize the beneficial effects of androgens on follicle growth and function. However, if androgen exposure exceeds the physiological concentration range for women, this feedback circuit can no longer provide sufficient protection, and adverse biological effects of excess androgens affect follicle growth and function. We describe a gradual, oocyte-centered process of follicle developmental arrest in our study. From this, we extrapolate that local androgen excess may negatively affect oocyte quality in PCOS, which in turn could co-orchestrate antral follicle arrest.

In conclusion, we have shown that androgen homeostasis in the developing preantral and antral murine follicle is crucial to ensure optimal growth, steroidogenesis, and oocyte maturation. Our study illustrates the dynamic steroid metabolome of the developing follicle *in vitro* and a feedback mechanism at the level of the isolated follicle that responds to androgen excess with downregulation of intrafollicular androgen production; these findings have translational implications for our understanding of PCOS and low ovarian reserve.
