# Supplementary material for: The Steroid Metabolome in the Isolated Ovarian Follicle and Its Response to Androgen Exposure and Antagonism
Source: Endocrinology. 2017 Feb 23;158(5):1474–85. doi: 10.1210/en.2016-1851 (PMC5460835; doi:10.1210/en.2016-1851)
Supplement: Supplementary file 1 [file en.2016-1851.st1.docx]

**Supplemental Appendix**

**Suppl. Table 1**

Relative mRNA expression of target genes of steroidogenesis, expressed as ΔCT values (normalized against 18S ribosomal RNA) ± SD (for each triplicate experiment from 18-30 follicles from each treatment group pooled prior to RNA extraction).

| Gene name | Day 0 | Day 6 | P (Day 0 vs. Day 6) |
| --- | --- | --- | --- |
| *star* | 15.4±0.4 | 15.3±0.7 | 0.8 |
| *cyp11a1* | 13.9±0.4 | 12.5±0.1 | **0.006** |
| *cyp17a1* | 13.3±0.05 | 12.8±0.07 | 0.4 |
| *cyp19a1* | 15.5±0.09 | 12.3±1.3 | **0.01** |
| *hsd3b1* | 10.9±1.4 | 9.6±0.3 | 0.2 |
| *hsd17b1* | 15.2±0.1 | 11.1±0.2 | **<0.0001** |

Abbreviations: steroidogenic acute regulatory protein (star), cytochrome P450 cholesterol side-chain cleavage (cyp11a1), cytochrome P450 17-hydroxylase (cyp17a1), 3β-hydroxysteroid dehydrogenase (hsd3b1), 17β-hydroxysteroid dehydrogenase (hsd17b1) and cytochrome P450 aromatase (cyp19a1).
